# Infrared Image Super Resolution by Combining Compressive Sensing and Deep Learning

**DOI:** 10.3390/s18082587

**Published:** 2018-08-07

**Authors:** Xudong Zhang, Chunlai Li, Qingpeng Meng, Shijie Liu, Yue Zhang, Jianyu Wang

**Affiliations:** 1 University of Chinese Academy of Sciences, Beijing 101408, China; shzhangxd@aliyun.com (X.Z.); 13122302160@163.com (Q.M.); liushijie163@163.com (S.L.); yuezh2015@163.com (Y.Z.); 2 Key Laboratory of Space Active Opto-Electronics Technology, Shanghai Institute of Technical Physics of the Chinese Academy of Sciences, Shanghai 200083, China; lichunlai@mail.sitp.ac.cn

**Keywords:** super resolution, infrared images, compressive sensing, deep learning, convolutional neural networks

## Abstract

Super resolution methods alleviate the high cost and high difficulty in applying high resolution infrared image sensors. In this paper we present a novel single image super resolution method for infrared images by combining compressive sensing theory and deep learning. Low resolution images can be regarded as the compressed sampling results of the high resolution ones in compressive sensing. With sparsity in this theory, higher resolution images can be reconstructed. However, because of diverse level of sparsity for different images, the output contains noise and loss of high frequency information. Deep convolutional neural network provides a solution to relieve the noise and supplement some missing high frequency information. By concatenating two methods, we manage to produce better results in super resolution tasks for infrared images than SRCNN and ScSR. PSNR and SSIM values are used to quantify the performance. Applying our method to open datasets and actual infrared imaging experiments, we also find better visual results are preserved.

## 1. Introduction

Nowadays high resolution (HR) images, possessing richer scene information and better visual quality than low resolution (LR) ones, are more desirable in many circumstances. However, the instrumentation limits make the HR images expensive and hard to achieve [1]. This problem is much more severe for infrared (IR) image sensors than visible (VIS) ones. Due to long wave-length, low resolution IR images always suffer from missing details including texture, contexture, edge information, etc. [2]. Less difficulty in optics and sensors manufacturing, super-resolution (SR) method is the most common task that widely used in many areas such as medical imaging [3], remote sensing [4], face recognition [5] and microscopy [6].

SR solutions are grouped into two categories: multi-frame SR (MFSR) and single-image SR (SISR) [7]. For MFSR, a sequence of LR images are captured to compose a HR image using the relative geometric and/or photometric displacements from the target HR image [8]. However, the necessary highly related sequences of images are not often available. In this paper, we focus on single image super resolution (SISR). As it is an inherently ill-posed problem, we have to rely on strong prior information to accomplish the task [9]. Sparsity based methods and learning based methods represent two typical ways of utilizing prior information [10].

Images, as is 2-D signal, exhibit sparsity in some domain, which enables Compressive Sensing (CS) theory to reconstruct the original HR images with LR ones with less sample rate. CS theory has already been proved to be effective and powerful in SR tasks [11]. Many SISR problems have been analyzed under different sparse bases, such as Wavelet [12], Discrete Cosine Transformation (DCT) [13] and Discrete Fourier Transform (DFT) [14]. Recently more practical applications reveal that a signal is more sparse with respect to an over-complete dictionary than a basis [15]. Besides, in order to accurately reconstruct the coefficients of the original signal in sparse domain, optimal reconstruction methods are needed. Different methods possess different performance, while we choose the iteratively reweighted least squares (IRLS) as the optimal reconstruction algorithm for its high reconstruction performance through experiments [16]. Its mechanism will be discussed later in this paper.

Apart from sparsity-based methods, learning-based ones also benefit from prior information. As deep-learning has recently prospered, many learning-based algorithms have been used in SISR, such as VGG [17], ResNet [18] and GAN [19]. Initially SRCNN [20] was the first 3-layered Convolutional Neural Network (CNN) utilized for SR tasks. Lately, for better performance the network structure goes much deeper. Besides, residual learning networks, when used in SR tasks, have been proved to possess better visual performance and Peak Signal to Noise Ratio (PSNR) performance.

In recent several years, many researchers have tried to combine CS and deep learning to produce better SR task solutions. Duan et al. [21] used deep learning to capture the image features and apply them to reconstruct HR images with the help of the sparsity in CS. Bora et al. [22] used generative models to replace the sparsity bases in CS and achieve satisfying results.

In this paper, we provide a novel combination architecture. We take advantage of sparsity in CS to recover the high frequency information in HR images. Then we build a deep-layer CNN to promote the performance of IRLS in CS. Residual learning [23] ensures that with our algorithm it is easier to optimize the results by denoising and reconstructing the output image of CS. By concatenating the two methods we achieve better performance than SRCNN [20] and ScSR [24] that utilize sparsity and a neural network alone. In simulations and actual infrared imaging experiments, we apply our method to IR images and we verify its performance both visually and quantitatively.

## 2. Super-Resolution Framework

### 2.1. Super-Resolution with Compressive Sensing Theory

CS theory combines sampling and compression into non-adaptive linear measurement process [25] at a rate significantly below the Nyquist [26]. The classical CS acquisition process can be depicted as:

(1) y=Φx=ΦΨs=θs.

Here $y\in {\mathbb{R}}^{M}$ is the vector of stacking measurements. $x\in {\mathbb{R}}^{N}$ (M<N) is the original compressible signal. Φ is the M×N measurement matrix and θ≔ΦΨ where Ψ is the N×N basis matrix. Vector s is the coefficients of x in the Ψ domain. Usually a Gaussian random matrix will be used as Φ. In SISR tasks, y will be regarded as the low projection of the HR image x, and Φ is corresponding to a downsample matrix in SISR [13]. Referring to the binning process of image sensors [27], we believe that one pixel in a LR image equals to the average of corresponding k×k neighbor pixels in HR one. Therefore Φ with M×N dimension, where $N/M=k^{2}$, should function as this downsampling process [28].

x in the spatial domain can be represented by vector s in the Ψ domain, which is K-sparse (K<N coefficients in s are non-zero). With sufficient sampling rate, s will be correctly recovered from Equation (1) by solving such an $l_{p}$-norm optimization problem:

(2) $$\underset{s}{\min}\frac{1}{2}{\left\|s\right\|}_{p}^{p},\;s.t.y=\Phi x=\Phi \Psi s=\theta s.$$

Ψ, the sparsity basis, has been widely proved validity using wavelet basis [29]. In our algorithm, we utilize DCT basis instead, because of its better performance under numerous experimental conditions. In this paper, we will use Peak Signal to Noise Ratio (PSNR) and structural similarity index (SSIM) to quantify the performance of the SR method. After testing on widely used 400 images [30] of size 180×180, we find that on average the DCT basis outperforms the wavelet basis by 14% higher in PSNRs and 26% higher in SSIMs.

Corresponding to the basis, the reconstruction algorithm is also important for our SR tasks. In order to solve this underdetermined equation finding accurate x, many optimization methods have been developed these years, such as Orthogonal Matching Pursuit (OMP) [31], Subspace Pursuit [32], Relevance Vector Machine (RVM) [33] and Iteratively reweighted least squares (IRLS) [16]. Iteratively reweighted least squares (IRLS) is selected for better visual and quantitative results, where p=1.

The IRLS method we use is based on solving (2) with modified objective function that at each iteration the function approaches $\overset{N}{\underset{k=1}{\sum }}{\left|s\right|}^{p}$ [27]. Simply, we substitute the ${𝓁}_{p}$ objective function in (2) with a weighted ${𝓁}_{2}$ norm:

(3) $$\underset{s}{\min}\overset{N}{\underset{i=1}{\sum }}w_{i}{s_{i}}^{2},\;s.t.y=\Phi x=\Phi \Psi s=\theta s.$$

where $w_{i}={\left|s_{i}^{\left(n-1\right)}\right|}^{p-2}$ is the first-order approximation to the ${𝓁}_{p}$ objective function. $w_{i}$ changes at each iteration until $w_{i}{s_{i}}^{2}$ is sufficiently close to ${\left\|s\right\|}_{p}^{p}$ in (4) after convergence. Then the solution of (3) is:

(4) $$s^{\left(n-1\right)}=Q_{n}\theta^{T}{\left(\theta Q_{n}\theta^{T}\right)}^{-1}y,$$

where $Q_{n}$ is the diagonal matrix with entries:

(5) $$1/w_{i}={\left|s_{i}^{\left(n-1\right)}\right|}^{2-p}.$$

The convergence criterion for each iteration stage can be depicted as:

(6) $$\frac{\left\|s^{n}-s^{n-1}\right\|}{1+\|s^{n-1}\|}<\frac{\sqrt{\mu }}{100}.$$

After (6) is attained, μ is reduced by a factor of 10, and the iterative procedure is repeated until $\mu <10^{-8}$ [34].

In conclusion, the HR image x can be depicted by sparse vector s in Ψ domain. The input of the algorithm, the original LR image, is regarded as the compressed measurements. Finally, x can be resolved with reconstruction algorithm. The detailed parameters in this algorithm are demonstrated in Algorithm 1.

**Algorithm 1.** IRLS Method for Super-Resolution**Parameters:**p = 1, use DCT basis as Ψ, down-sampling matrix Φ, *N*/*M* = 2 or 3, μ=1.**Step 1:** Initialize the size of output image and the formation of sparsity basis. **Step 2:** Do the inner loop:**2.1** Initialize n≔1, $s_{0}=\left(0,0,\ldots 0\right)$ and $Q^{\left(0\right)}$ = O.**2.2** Update $Q^{\left(n\right)}$ using (5).**2.3** Compute $s^{n}$ using (4).**2.4** If (6) is satisfied, go to step 3; otherwise, let n=n+1 and go to step 2.2.**Step 3:** Update the regularization parameter, μ=μ/10.**Step 4:** If $\mu <10^{-8}$, finish; else, go to Step 2.

### 2.2. Image Denoising and Reconstruction with Deep Learning

**Figure 1:**
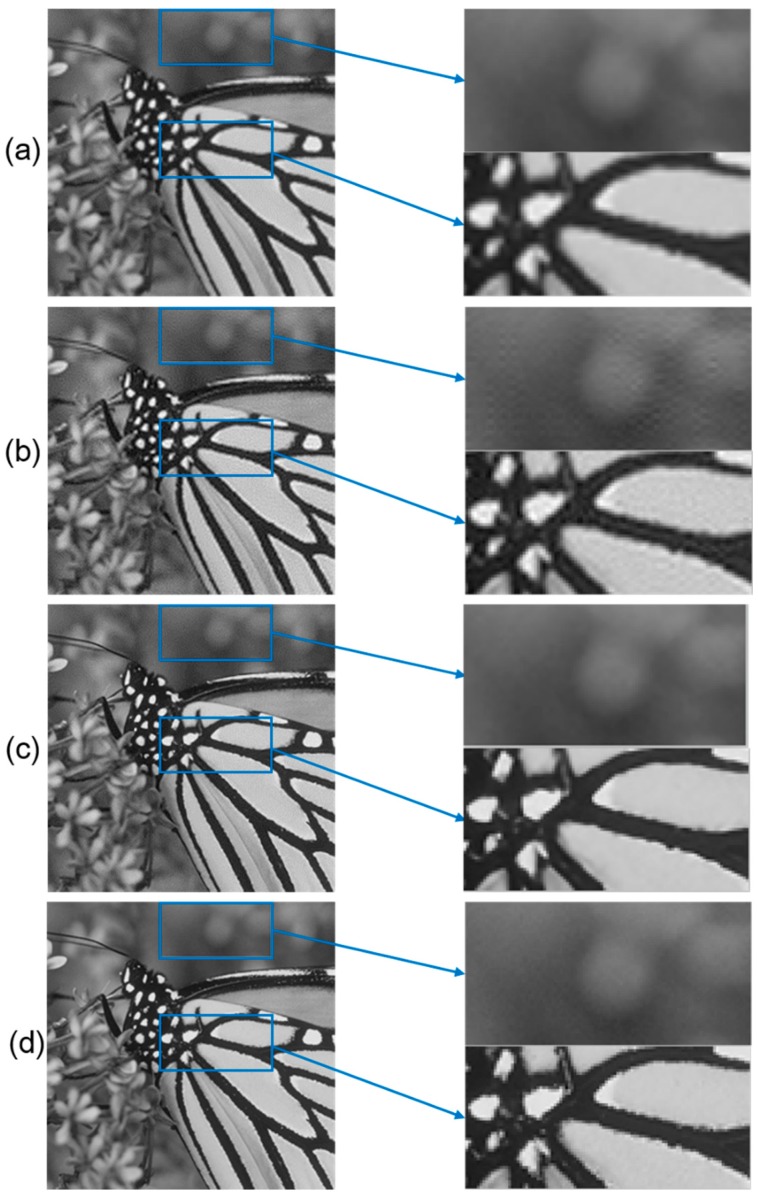
Illustration of fixed pattern noise in CS method when upscaling factor is set 2 for demonstration. Subfigure (**a**) is the output of bicubic SR method; subfigure (**b**) is the output of CS; subfigure (**c**) is the reconstructed output of CNN; subfigure (**d**) is the original HR image. The corresponding zoomed pictures are placed on the right. The PSNR and SSIM of CS are 28.86 dB and 0.8950. The PSNR and SSIM of bicubic are 28.22 dB and 0.9260. The PSNR and SSIM of CNN are 34.18 dB and 0.9719.

Practically, it is hard to find an absolutely correct y, which represents the HR image in SR tasks. Mostly the algorithms will come to a local optimal solution that makes the output images contain fixed pattern noise, which is illustrated in Figure 1. By comparing the output of CS, bicubic method and the original HR image, visually we find that CS preserves more texture information and less blur effect, but contains some fixed pattern noise. After using our CNN, the noise is visually alleviated. Although the PSNR of CS output is 0.64 dB higher than bicubic, the SSIM of CS is 0.031 lower. As SSIM calculates the covariance value of the images representing the structural information of the objects in images [35], studies show that it is more vulnerable to fixed-pattern noise than pixel difference-based measurement, PSNR [36]. Therefore a method that protects the high spatial frequency information while wiping out the fixed pattern noise is necessary. After using our CNN, the structure of which will be discussed in the following paragraph, the PSNR is increased to 34.18 dB and the SSIM is increased to 0.9719. These values proves that our CNN is effective in denoising and reconstruction.

From the results, we believe that the CNN not only deals with the fixed pattern noise, but also helps supplement more high frequency information. As the images change, the level of sparsity changes as well. Some HR images may contain more high frequency information that won’t be recovered by a certain sparsity basis, causing the limits of the CS method, which means using CS alone won’t recover all the high frequency information. In that case, we also need more efforts to supplement the missing information during the SR process. Deep learning with powerful image processing ability has been applied to many tasks like image denoising, demosaicing [37] and reconstruction [38]. Zhang et al. [29] designed a deep convolutional neural network (CNN) for image Gaussian denoising, which is called DnCNN. Residual learning and batch normalization greatly benefit its performance. Inspired by DnCNN, we modified its network architecture to accomplish the denoising and high frequency information supplementation in our SR tasks.

**Figure 2:**
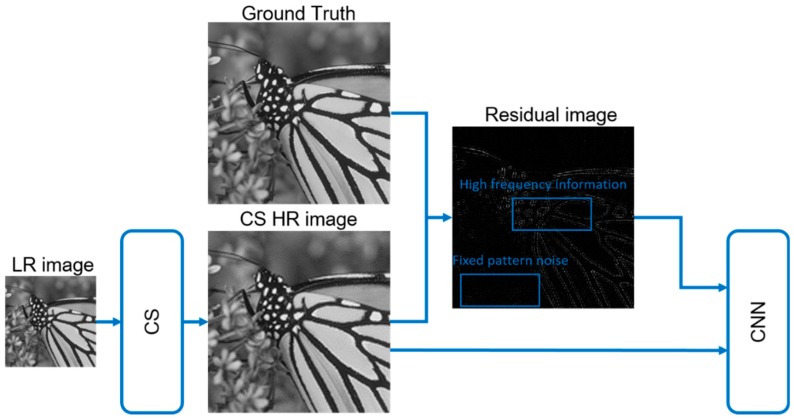
The residual learning progress of our method.

The most essential part of our CNN model is the residual learning. Although the output of CS contains fixed pattern noise, we are not able to describe its formation with a designed rule in order to eliminate it. However deep learning provides us with trainable convolutional filter, in which case the noise of each HR image can be detected and eliminated after training the CNN model. Residual learning enables us to train each layer of CNN to fit the residual mapping instead of the original image. Formally, we denote the HR output of CS as H(j), and the original HR image, which is the ground truth, as G(j). Here j denotes the index of each image. The residual image R(j)=G(j)−H(j), represents the fixed pattern noise of each image. Researches have revealed that residual image is easier to be optimized by CNN [23]. Figure 2 shows the proposed SR architecture when training.

The target of our CNN is to estimate the residual image of every CS output for promoting the performance. The averaged mean square error between estimated residual image and the true residual one:

(7) $$l\left(\Theta \right)=\frac{1}{2N}\overset{N}{\underset{i=1}{\sum }}{\left\|\tilde{R}\left(\Theta ,i\right)-R\left(\Theta ,i\right)\right\|}^{2},$$

denotes the loss function to learn the trainable parameters Θ in CNN. Corresponding to ith training image, $\tilde{R}\left(\Theta ,i\right)$ represents the estimated residual image produced by our CNN, while R(Θ,i) represents the true residual image used for training.

**Figure 3:**
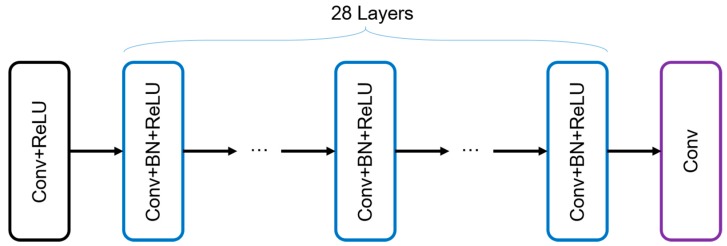
The architecture of the CNN.

Researches reveal that the depth of network is of great importance for better results [23]. Therefore, we challenge to modify the CNN into a deeper network with 30 layers. Inspired by DnCNN, our network consists of three types of layer, which is shown in Figure 3.

In the first layer, we utilize 64 filters of 3×3 size as the convolution kernels to generate 64 feature maps. And rectified linear units (ReLU, max(0,·)) are utilized as the nonlinear activation function for speeding up the optimization. The 28 hidden layers are of the same formation. 64 filters of size 3×3×64 are connected with batch-normalization (BN) [39] in the hidden layers for accelerating training speed. For the last layer, a 3×3×64 convolution is used for reconstructing the residual image.

After simulation experiments, we find that Adaptive Moment Estimation (Adam) optimization [40] algorithm outperforms Stochastic Gradient Descent (SGD) [41]. Therefore, we choose Adam as the optimization method for our CNN. Adam is a first-order gradient-based optimization algorithm, which is based on adaptive estimates of lower-order moments of the gradients. The pseudo-code is shown in Algorithm 2.

**Algorithm 2.** Adam Method for Optimization**Parameters:**α is the stepsize; $\beta_{1},\beta_{2}\in [0,1),\lambda \in [0,1)$ are the exponential decay rates for the moment estimates; l(Θ) is the loss function with parameter Θ.**Step 1:** Initialize the parameters as $\beta_{1}=0.9$, $\beta_{2}=0.999$, $\lambda =1-10^{-8}$, α=0.001.**Step 2:** Initialize the vectors.$m_{0}\leftarrow 0$ is the initial first moment vector.$v_{0}\leftarrow 0$ is the initial second moment vector.t←0 is the initial timestep.**Step 3:** Do the inner loop: **3.1**t←t+1*.* Update the timestep.**3.2**$\beta_{1,t}\leftarrow \beta_{1}\;\lambda^{t-1}$. Decay the first moment running average coefficient.**3.3**$g_{t}\leftarrow \nabla_{\theta }f_{t}\left(\theta_{t-1}\right)$. Get gradients corresponding to loss function at timestep *t*.**3.4**$m_{t}\leftarrow \beta_{1,t}\cdot v_{t-1}+\left(1-\beta_{1,t}\right)\cdot g_{t}$. Update biased first moment estimate. **3.5**$v_{t}\leftarrow \beta_{2}\cdot v_{t-1}+\left(1-\beta_{2}\right)\cdot g_{t}⊙g_{t}$. Update biased second raw moment estimate. **3.6**$\hat{m_{t}}\leftarrow m_{t}/\left(1-\beta_{1,t}\right)$. Compute bias-corrected first moment estimate.**3.7**$\hat{v_{t}}\leftarrow v_{t}/\left(1-\beta_{2}\right)$. Compute bias-corrected second raw moment estimate.**3.8**$\Theta_{t}\leftarrow \Theta_{t-1}-\alpha \cdot \hat{m_{t}}/\left(\sqrt{\hat{v_{t}}}+\epsilon \right).$ Update parameters, where ϵ is for preventing the denominator to be zero. **3.9** if $\Theta_{t}$ is converged, go to step 4; otherwise go to step 3.1. **Step 4:** Return $\Theta_{t}$.

Most parameters of Adam are set the same as the ones in [40], as the mini-batch size is 128 and the learning rate decays exponentially from 1 × 10^−1^ to 1 × 10^−4^ during 50 epochs of training.

**Figure 4:**
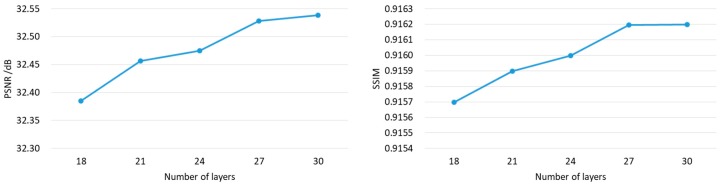
The average PSNR & SSIM upscaling factor 2 & 3 versus different layers CNNs under 50-epoch training when applied to the 12-image test set.

We use the MatConvNet package in Matlab 2017a to train our CNN. A Intel^®^ Core^TM^ i5-4670k CPU operating at 3.4 GHz and an Nvidia 1080Ti GPU are used. Experiments show that the deeper the network goes, the better PSNR performance becomes, as is shown in Figure 4. However, for a deep network of 30 layers and 128 mini-batch size, a great burden has been placed on the GPU memory. 30 layers with 128 mini-batch size is up to the limit of the GPU memory.

### 2.3. The Whole Super-Resolution Algorithm Architecture

**Figure 5:**
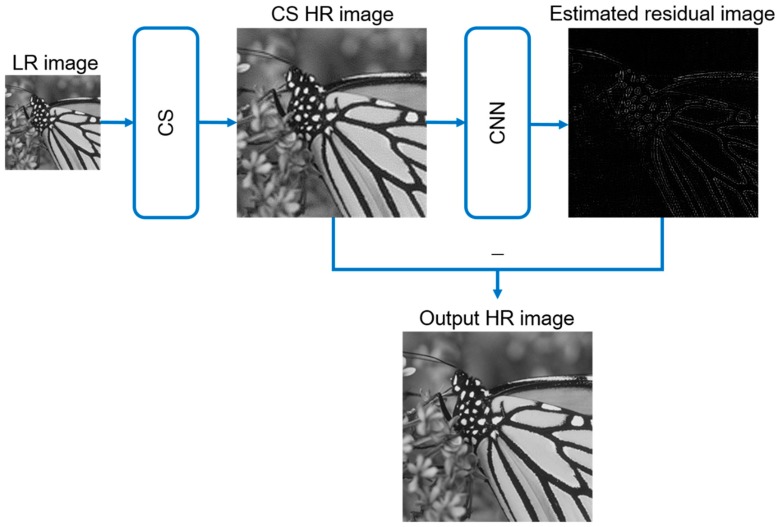
The whole architecture of the proposed SR method.

In Figure 5 we show the whole architecture when using the proposed method to accomplish the SR target. After training process, the CNN is used to eliminate the fixed pattern noise in the output of CS SR method and supplement some high spatial frequency information to it.

## 3. Simulation Results

Before applying our method to real scenes captured by infrared sensors, we test it with some open datasets by comparing it with SRCNN [20] and ScSR [24] that utilize sparsity and neural network alone. Considering that there are not enough open image data sets for training at infrared wavelengths, we choose widely used 400 VIS images [29] of size 180×180 as the training dataset. The experimental results show that the model trained by VIS dataset functions well when dealing with IR images. A larger training dataset is more preferable, but leads to more training time pressure. After testing we find that 400 images are enough to get high performance, and the training time is acceptable. About 10 h for training is needed for our CNN. This trained model in VIS is used for super resolution tasks in VIS images and IR images.

**Figure 6:**
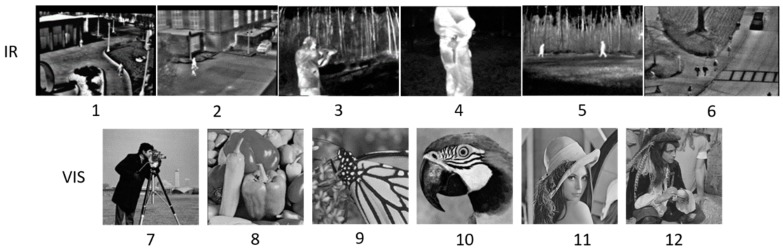
The 12 IR and VIS images used for performance evaluation.

We apply our method to six infrared images collected from the OSU thermal pedestrian database, OSU Color and Thermal Database and Terravic Motion Infrared Database of the OTCBVS dataset collection [42]. Besides, we also apply our method to six widely used VIS images to prove the robustness. Figure 6 shows the overview of the 12 total images regarded as the test set. It is worth highlighting that the training set should not share the same images with the test set in order to avoid a logical paradox. Therefore the 12 IR and VIS images are not included in the 400 images for training.

In this paper the upscaling factors are set 2 and 3. We down-sample the HR image into two LR one by merging 2×2 or 3×3 neighbor pixels on average in order to simulate two kinds of LR images. We compare the SR images with the original HR ones by quantifying the performance in PSNR and SSIM, the results of which is shown in the Table 1 and Table 2. Besides the execution time is also provided in the tables for considering the complexity of our algorithm.

Before discussing the SR reconstruction performance, the execution time of three methods also attracts great interest. SRCNN exhibits the least time consumption, while ScSR and our algorithm need far more execution time. Most time of our algorithm is spent on solving the optimization problem for compressive sensing architecture in (2). This is because the time complexity of IRLS is high, despite its better accuracy in reconstruction. Another fact that draws great attention is that our algorithm needs far less time for SR of upscaling factor of 3 than of 2, unlike ScSR and SRCNN. The reason is that LR images produced by merging 3×3 neighbor pixels from HR ones contain lower spatial resolution and less amount of information than those produced by merging 2×2 pixels, which means fewer constraint conditions in (3) and fewer dimensions of vector s in the Ψ domain in (3). After fewer iterations, IRLS will comes to the nearly accurate answers to get HR estimation. Therefore, we may predict that for even larger upscaling factors, our algorithm may perform much better in execution time.

**Figure 7:**
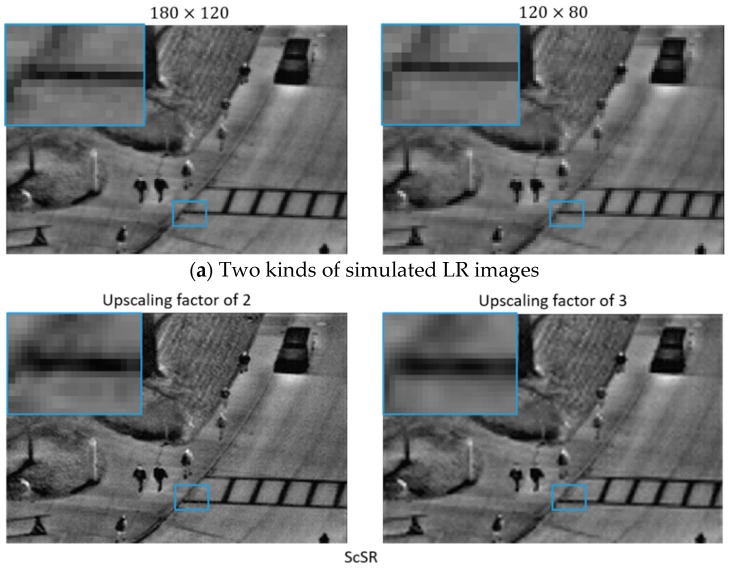

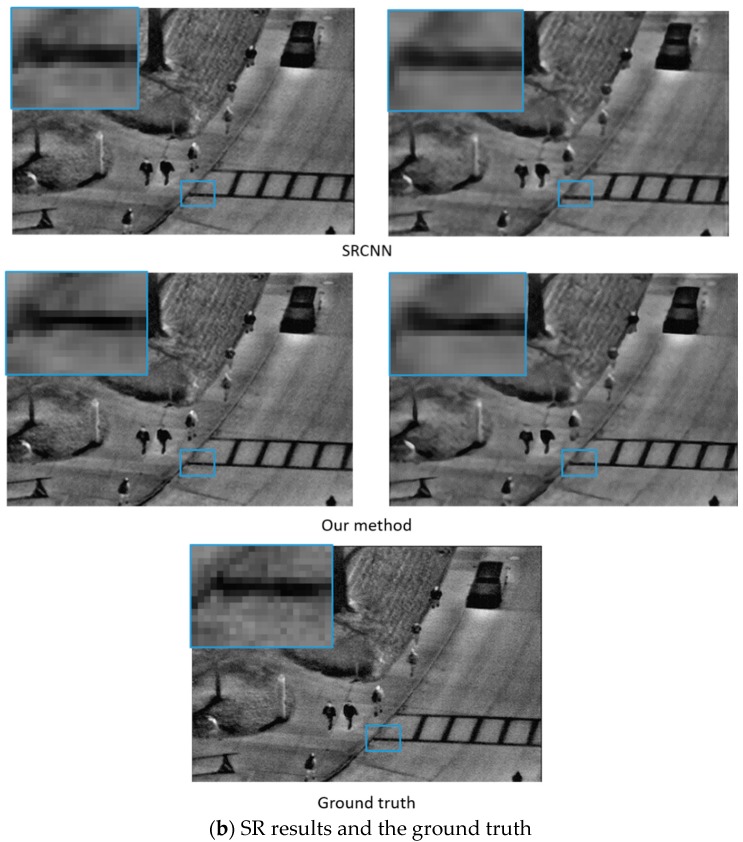
Visual comparison of SRCNN, ScSR and the proposed method with upscaling factors of 2 and 3.

We find that the proposed method has great advantages in PSNR values, while performing a little better than SRCNN and ScSR in SSIM values. We choose image 6, the infrared surveillance, in the test set as an example to show the performance visually. Figure 7 illustrates the visual comparison of three methods. The original HR image is of 360×240 pixels. After down-sampling, two kinds of LR image images are produced, which are of 180×120 pixels and of 120×80 pixels. We produce the SR images with SRCNN, ScSR and our method. The zoomed HR images are placed on the right.

The whole images comparison provides us the overall perception of different methods, where the texture feature in our method appears to be clearer. Moreover, in our results, the surroundings near the objects are of less distraction and less noise. From the zoomed images, we find that the edges in the output of our method are more distinct. In details, the contours of the zebra crossing in our method possesses higher fidelity and higher contrast compared to the one in SRCNN and ScSR. We believe this advantage may help a lot in further image recognition tasks.

## 4. Imaging Experiments

**Figure 8:**
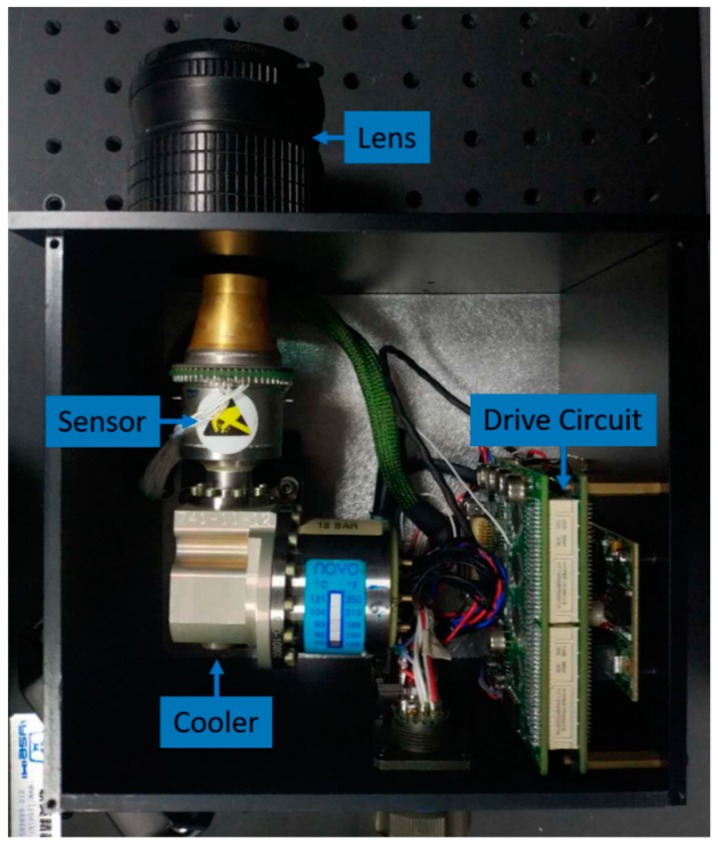
The imaging structure of our infrared system. MARS-VLW-RM4 from Sofradir Company is the infrared image sensor of 320×256 resolution. The lens is suitable for Very Long-Wave band of f=60 mm and F=2.0 from Lenstech Company in Beijing, China.

In this section, we apply our method to an infrared image sensor to testify the portability and generality. As demonstrated in Figure 8, we use MARS-VLW-RM4 from the Sofradir Company (Palaiseau, France) as the infrared image sensor, whose original resolution is 320×256. Its sensitivity to infrared radiation in the Very Long-Wave band (8–12 μm) make ensure its applicability for military and civilian surveillance purposes. However due to the high cost of manufacturing, it is difficult to increase the resolution. Using CS theory and deep learning, we are able to produce higher resolution infrared images without changing the original sensor.

**Figure 9:**
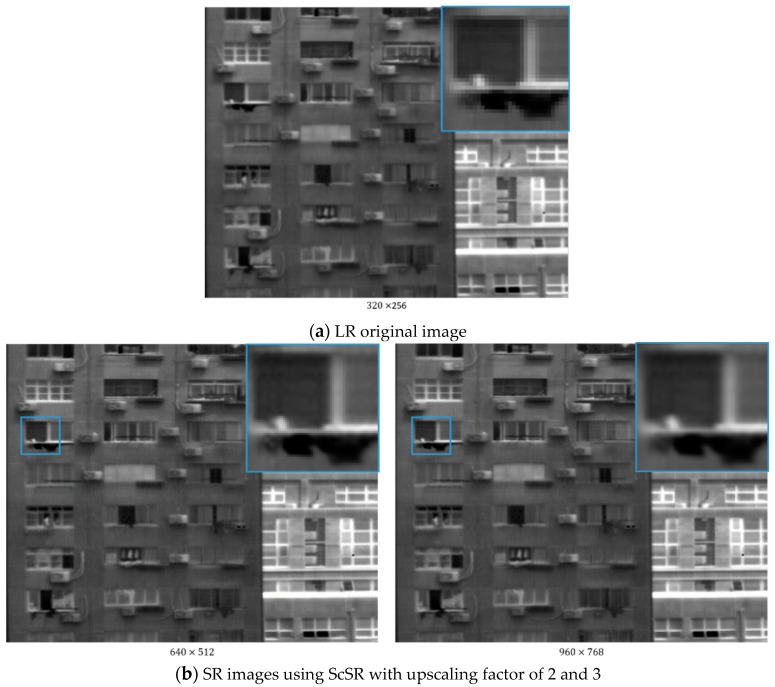

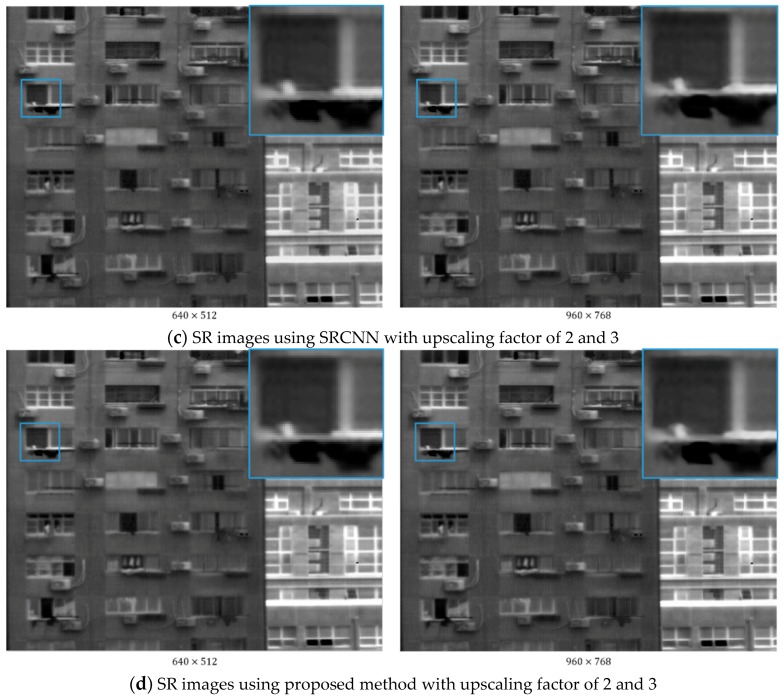
Imaging results comparison of the SR output.

The parameters and trained models are the same as the ones in the simulation section. As lack of ground truth for HR infrared images, the performance will be judged visually in this section. With upscaling factor of 2 and 3, we will produce HR images of 640×512 and 960×768 resolution. The results are shown in Figure 9.

Visual comparison between the LR and HR images and the 3 different methods demonstrate the advantage of our method. In HR ones, image details, like textures and contours, are more sufficient and mosaic effects caused by LR image sensor are relieved. Therefore, higher resolution infrared images which surpass the original image sensor’s resolution are available by using our method. Moreover, compared to zoomed images of ScSR, our results contain less blur and sharper features. As to SRCNN, its reconstruction noise of the windowsill in the zoomed images shows its inferiority to our method.

## 5. Conclusions

In this paper we present a novel super resolution method that is the combination of compressive sensing theory and deep learning. Our method consists of two parts. The first one utilizes the spatial sparsity of CS theory to reconstruct a HR image which contains higher frequency information. The second part uses the trained network to remove the fixed pattern noise that was introduced in the first part and supplement some additional high frequency information which is learnt from the training set. Its high performance helps us to acquire higher resolution infrared images without suffering from the high cost and difficulty in applying large infrared sensors. The performance has been demonstrated visually and quantitatively in the simulation tasks. Our method possesses better performance with higher PSNR and SSIM values than SRCNN and ScSR in both visible and infrared datasets. We apply our method to a Very-Long-Wave band infrared sensor to testify its portability and generality. With low resolution infrared sensor, we are able to produce higher resolution images.

As our work only analyzes the monochrome images, we expect more studies will focus on spectral images’ super-resolution problems.

## Figures and Tables

**Table 1:** SR results with upscaling factor of 2.

Image	SRCNN			ScSR			Proposed Method		
	PSNR	SSIM	Time/s	PSNR	SSIM	Time/s	PSNR	SSIM	Time/s
1	33.03	0.9529	2.5	33.29	0.9662	17.6	34.78	0.9629	9.6
2	36.78	0.9633	1.6	36.34	0.9685	17.7	38.08	0.9689	12.1
3	34.42	0.9700	1.7	34.26	0.9718	21.1	34.65	0.9702	15.2
4	40.59	0.9769	1.6	41.36	0.9793	20.9	41.53	0.9786	15.4
5	35.34	0.9652	1.7	35.93	0.9691	20.9	36.10	0.9678	15.2
6	30.63	0.8118	1.8	30.34	0.8141	24.4	31.08	0.8154	14.1
7	28.20	0.9005	1.5	27.56	0.8940	17.7	29.48	0.9111	14.5
8	32.66	0.9398	1.4	31.75	0.9333	17.3	33.72	0.9464	53.0
9	32.51	0.9618	1.5	30.84	0.9520	16.7	34.18	0.9719	57.6
10	28.55	0.9180	1.5	28.41	0.9169	17.9	29.59	0.9254	57.2
11	36.19	0.9381	9.8	35.84	0.9353	70.1	36.74	0.9380	56.8
12	32.98	0.9201	10.2	32.44	0.9147	69.3	33.55	0.9265	54.0

**Table 2:** SR results with upscaling factor of 3.

Image	SRCNN			ScSR			Proposed Method		
	PSNR	SSIM	Time/s	PSNR	SSIM	Time/s	PSNR	SSIM	Time/s
1	28.22	0.8666	1.8	27.42	0.8851	47.6	28.73	0.9001	5.0
2	32.06	0.9222	1.4	31.50	0.9194	51.3	32.42	0.9259	4.2
3	29.47	0.9045	1.5	28.36	0.9070	52.2	29.27	0.9117	4.2
4	36.59	0.9452	1.4	35.61	0.9542	53.5	37.30	0.9528	4.0
5	30.93	0.9011	1.5	30.89	0.9096	52.5	30.57	0.9045	4.8
6	28.48	0.7134	1.6	27.97	0.7127	60.5	28.72	0.7216	5.1
7	26.53	0.8427	1.3	26.11	0.8342	45.2	27.24	0.8596	5.1
8	30.44	0.9117	1.2	28.69	0.8977	43.0	31.18	0.9269	18.7
9	29.04	0.9105	1.3	26.94	0.8835	44.8	30.27	0.9334	22.3
10	26.12	0.8693	1.3	25.75	0.8640	46.0	27.08	0.8838	22.4
11	33.40	0.9097	9.2	32.66	0.9041	183.3	33.48	0.9124	22.7
12	30.79	0.8636	9.8	30.14	0.8543	186.7	31.17	0.8731	22.4
